# Masking of Figure-Ground Texture and Single Targets by Surround Inhibition: A Computational Spiking Model

**DOI:** 10.1371/journal.pone.0031773

**Published:** 2012-02-29

**Authors:** Hans Supèr, August Romeo

**Affiliations:** 1 Institute for Brain, Cognition and Behavior, Barcelona, Spain; 2 Department of Basic Psychology, Faculty of Psychology, University of Barcelona, Barcelona, Spain; 3 Catalan Institution for Research and Advanced Studies, Barcelona, Spain; University of Salamanca- Institute for Neuroscience of Castille and Leon and Medical School, Spain

## Abstract

A visual stimulus can be made invisible, i.e. masked, by the presentation of a second stimulus. In the sensory cortex, neural responses to a masked stimulus are suppressed, yet how this suppression comes about is still debated. Inhibitory models explain masking by asserting that the mask exerts an inhibitory influence on the responses of a neuron evoked by the target. However, other models argue that the masking interferes with recurrent or reentrant processing. Using computer modeling, we show that surround inhibition evoked by ON and OFF responses to the mask suppresses the responses to a briefly presented stimulus in forward and backward masking paradigms. Our model results resemble several previously described psychophysical and neurophysiological findings in perceptual masking experiments and are in line with earlier theoretical descriptions of masking. We suggest that precise spatiotemporal influence of surround inhibition is relevant for visual detection.

## Introduction

In perceptual masking, a target stimulus is rendered less perceptible or even invisible through the presentation of a second stimulus, the mask. Masking is therefore an important tool for understanding the neural mechanisms underlying visual perception. Of particular interest for our current study are neurophysiological experiments on figure-ground (FG) segmentation. FG activity segmenting the figure from background elements, is tightly linked to the visual experience of a sensory stimulus [1]–[6], and is believed to represent a neural correlate of phenomenal awareness [7], [8].

In the visual cortex contextual influences on neuronal activity have been interpreted as the neural substrate of FG segmentation where feedback projections from higher visual areas to lower areas provide the contextual information, e.g. [7]. This is in line with a reduced FG modulation after removal of cortical feedback to V1 [9]. Feedback may act as an attention mechanism to enhance the FG signal [10] that may have a feedforward origin [11]. In contrast to FG textures, the visibility of simple targets does not necessitate feedback [12], [13]. Backward masking that specifically blocks FG responses in monkey [14] and human [15] visual cortex is believed to be an effect of the disruption of recurrent or reentrant processing. Recently we described a simple model, based on spiking neurons that is able to perform figure-ground segregation in a purely feedforward manner [11]. According to the model results, feedforward segregation of figure from ground is robust and occurs independently of figure size contrast, and number. In the current study, we tested whether backward masking disrupts feedforward FG segregation.

Besides backward masking of figure-ground textures, a single target can be made invisible by the presentation of a surrounding mask (metacontrast-masking), if it immediately precedes (forward masking) or follows (backward masking) the target stimulus [16]. Similarly, masking occurs when two targets are sequentially presented at the same location (repetition masking). In repetition masking the second of two targets cannot be detected or identified when it appears close in time to the first [17]. It is argued that masking of a single visual target is caused by lateral inhibition [12], [18], [19]. To further provide supporting evidence for this idea we therefore tested our model using the above mentioned masking paradigms.

The findings of our masking experiments show that the model behavior bears resemblance to several previously described neurophysiological and psychophysical effects of masking. Our results are explained by the interference of surround inhibition by the mask. Moreover, our model data indicates that rebound spiking and phase resetting are important factors for explaining masking results. Based on our observations we suggest that FG masking is not specific to the interruption of feedback processing and that spatiotemporal influence of surround inhibition is relevant for visual detection.

## Results

### Figure-ground segregation

**Figure 1:**
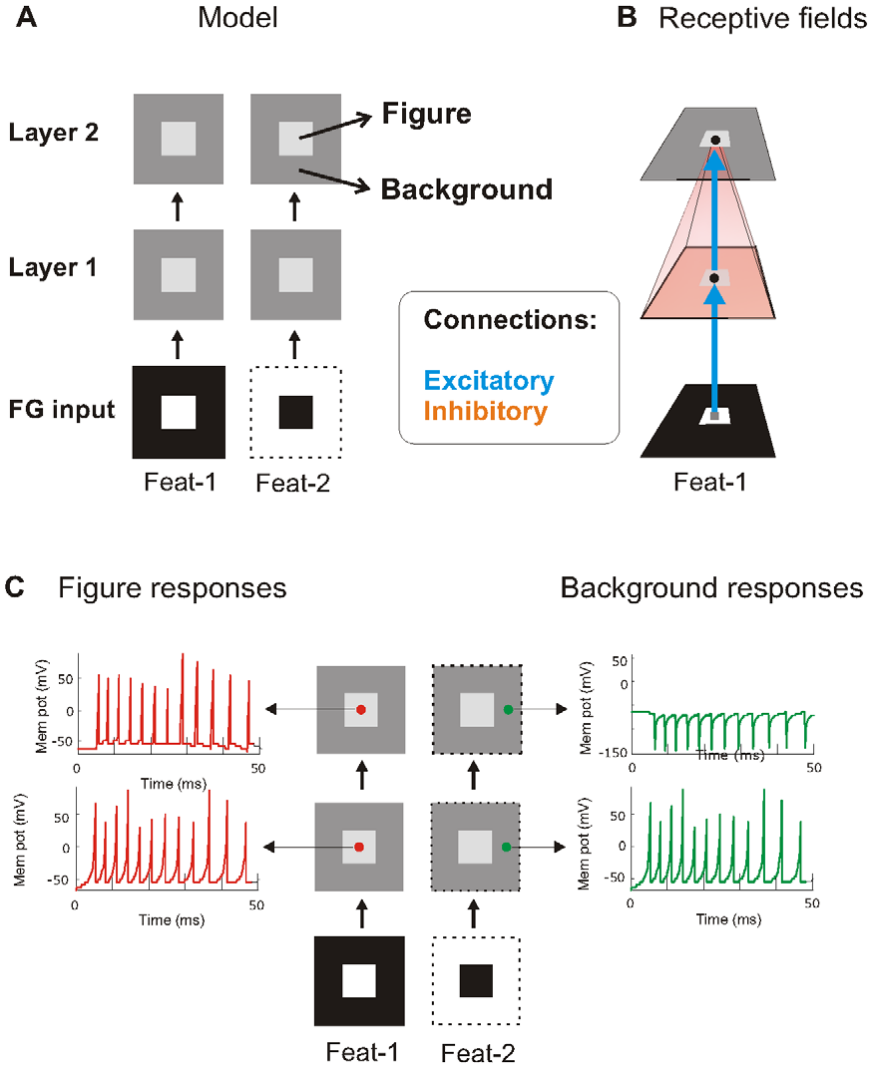
Model, receptive field organization and figure-ground segregation. **A**: The model consists of two separate feature channels (Feat-1 and Feat-2) each with two layers, which are unidirectionally connected (arrows). The white regions in the two lower squares indicate the stimulus input (FG input). Black regions provide no input to the model. In the two layers of the model, the light grey central squares depict the figure region and dark grey regions the background. **B**: Layer-1 neurons have a centre receptive field, i.e. they are driven by one input pixel. Layer-2 neurons have an excitatory centre and inhibitory surround receptive field. The central small black circles represent a neuron in the first and second layer of the model. The small grey square represents one input pixel. Blue arrows indicate point-to-point (retinotopic), excitatory connections and orange region represent the inhibitory connections from layer 1 to layer 2. **C**: Spike responses of the neurons in the first and second layer to figure-ground stimulus. Arrows point to the responses of neurons (small circles) lying on the figure (red traces) and background (green traces) regions.

We developed a 2-layered model of spiking neurons [11] using an input design (fig. 1a) that has been previously applied for modeling FG segregation [20]. The model consist of two feature channels (Feat-1 & Feat-2), which represent two separate neuronal cell populations with opposite preference for a single feature. Neurons in layer 1 transformed by means of their point-to-point excitatory connections (fig. 1b) the FG input into a spike map. These neurons responded within 50 ms with a transient burst of 12 spikes (lower red and green traces in fig. 1c). The layer-2 neurons integrated this information through local excitation and surround inhibition (fig. 1b). In the first feature channel (Feat-1), neurons at the centre location (figure) produced a similar spike burst as layer-1 neurons (fig. 1c, upper red trace). In contrast to the Feat-1 condition, neurons in the second feature channel (Feat-2) became quiescent (fig. 1c, upper green trace). Here the relatively large activated surrounding (background) region provoked a strong suppression neutralizing the point-to-point excitation of each neuron. This agrees with early studies reporting that neurons in early visual areas generally do not respond to large areas of uniform luminance. At longer times scales, however, responses of neurons located at the background were observed without affecting FG segregation [10], which agrees with reports showing that some V1 neurons do respond to uniform surfaces covering their RF, e.g. [21]. Also strong surround inhibition may produce rebound spiking at the figure location in the Feat-2 channel [11], [22]. In conclusion, in the second layer basic FG segregation by surround inhibition was achieved [11], [22], see also [23]; neurons located in the central figural region fired spikes while surrounding (background) neurons were silent.

### Figure-ground masking

**Figure 2:**
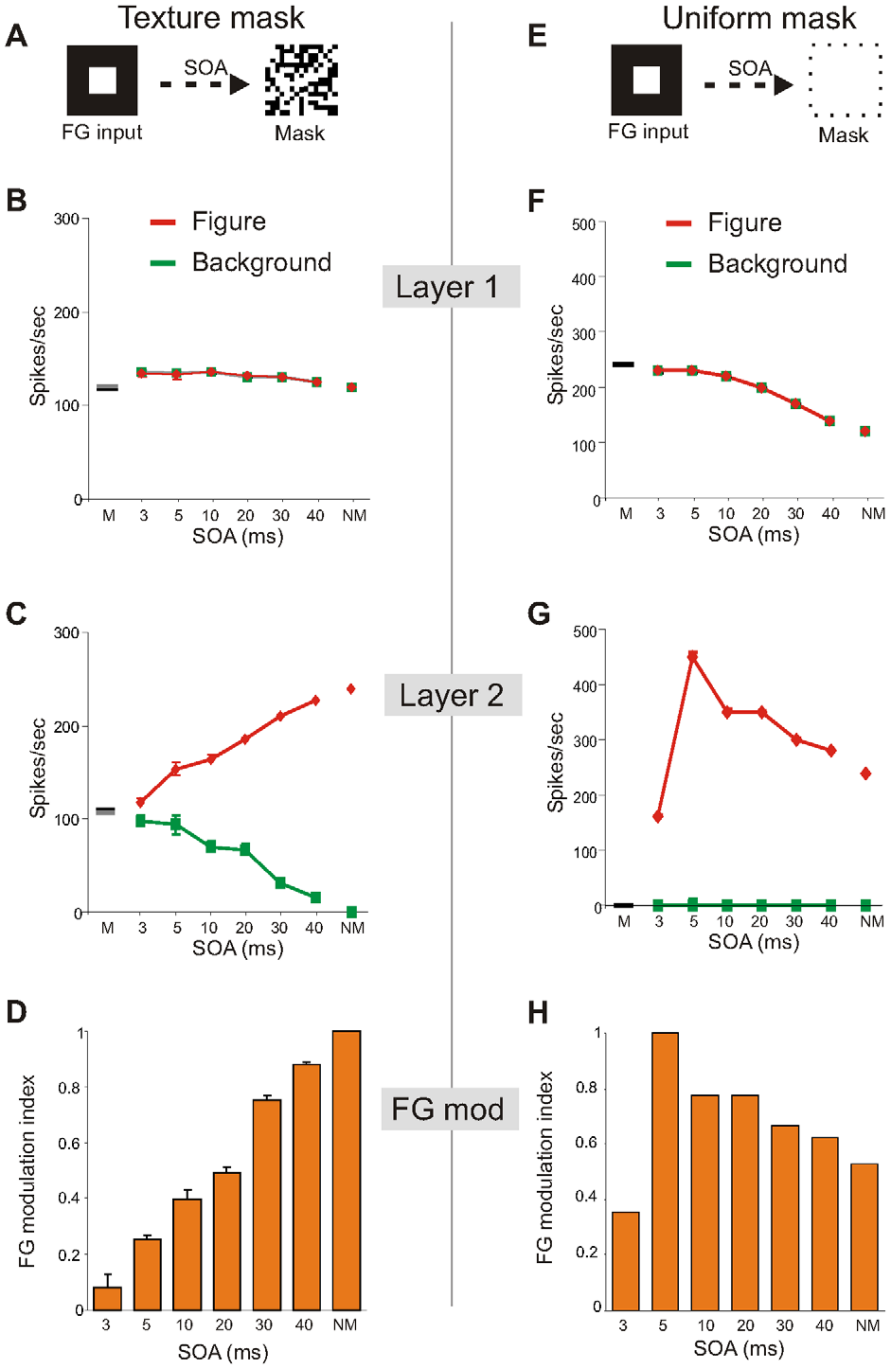
Masking of figure-ground signal. **A,E**: Left squares indicate the figure-ground stimulus, and the right one the pattern (A) or uniform mask (E). **B–G**: Average spike responses of the neurons in the first (B,F) and second (C,G) layer to figure-ground stimulus after pattern (B,C) and uniform (F,G) backward masking at different SOAs. **D,H**: Figure-ground modulation index at the different SOAs in the pattern (D) and uniform (H) mask condition. Time is from figure-ground stimulus onset. M is response to mask only and NM response to figure-ground stimulus only. Errors bars are SEM due to response variation by random pattern mask. Randomness is not present with a uniform mask.

To disrupt the FG signal we presented a pattern mask (fig. 2a; methods) at different variable times (Stimulus Onset Asynchronies, SOA) directly after presenting the FG stimulus. The backward mask had little effect on the firing rate of the neurons in the first layer (fig. 2b). At most we recorded a small increase in the firing rate for short SOAs compared to the responses to the FG stimulus without masking (NM) or to the responses to the mask alone (M; fig. 2b). Masking, however, had a strong effect on the firing patterns of the neurons in the second layer. At short SOAs responses were strongly suppressed for neurons at the central location (fig. 2c). Compared to the responses to an unmasked stimulus, firing rate dropped about 50% (240 spikes/sec vs 117 spikes/sec). By contrast, spike responses to the surround stimulus (background) increased with shorter SOAs reaching the same level as the figure responses (fig. 2c). Translating these figure and background responses into a modulation index showed that the segregation of figure from ground weakened for shorter SOAs and almost completely disappeared for the shortest SOA (fig. 2d). The increase and decrease of surround feedforward inhibition in the Feat-1 and Feat-2 condition, respectively explains the disappearance of FG modulation.

In contrast to a pattern mask, a uniform mask does not disrupt the figure ground signal nor does it impair visual perception of the figure [14]. We therefore replicated our masking experiment with a uniform mask (fig. 2e; methods). In the uniform mask condition, responses in the first layer increased substantially for shorter SOAs in both Feat conditions (fig. 2f). In the second layer, the input of layer 1 resulted in a large difference between the response rates of Feat-1 and Feat-2 conditions (fig. 2g). The central (figure) responses increased for shorter SOAs (except for the shortest SOA) while surrounding (background) neurons remained silent. As a consequence, the figure remained segregated (fig. 2h). Thus strong surround inhibition produced by the uniform mask did not abolish FG activity

**Figure 3:**
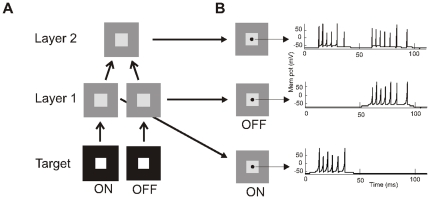
Model for metacontrast and repetition masking experiments. **A**: The first layer consists of two separate channels containing neurons that respond either to the onset (ON) or to the removal (OFF) of the target stimulus. Layer 2 integrates the input from layer 1. Receptive fields as in figure 1. **B**: Spike responses to the onset and removal of the target stimulus from units shown by small black circles.

**Figure 4:**
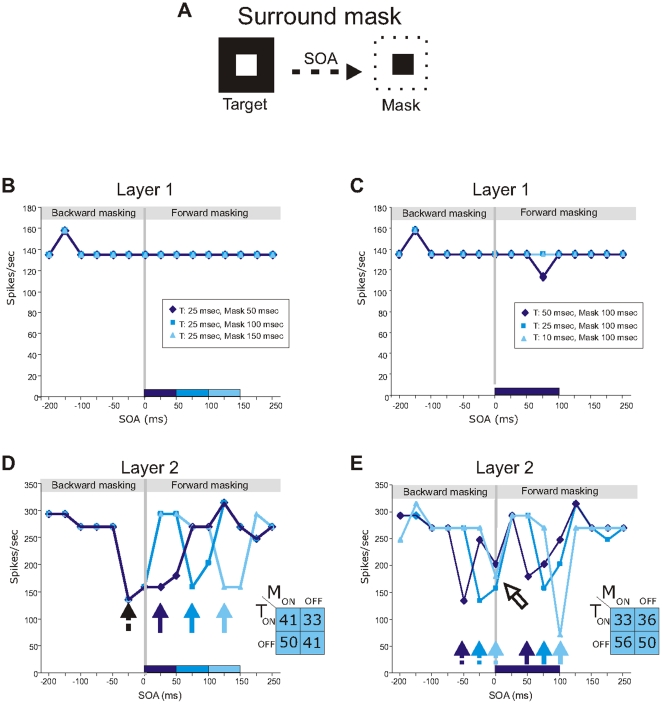
Metacontrast-masking results. **A**: Squares indicate the target input (left) and mask (right). White regions of the squares depict the input regions and black regions depict regions that provide no input to the model. **B–E**: Average spike responses of the neurons in the first (B,C) and second (D,E) layer to target masked at different SOAs in the metacontrastmasking experiment. Target responses are aligned on the time of mask onset. Filled arrows point to masking when timing of target offset and the mask removal coincides. Dashed arrows point to masking at concurrent target offset with mask onset. Time is from mask onset. Insets in (D,E) show the average percentage decrease in target responses. T is target and M is mask.

Besides a FG stimulus, a single target can be made invisible by metacontrast-masking, which only affects the surround of the target. To further test the role of surround inhibition in masking we examined the model behavior after metacontrast masking (see methods). Neurons in the both layers responded with a transient burst to the presentation (at t = 0 ms) and removal (at t = 50 ms) of a target stimulus (fig. 3b). We then briefly presented the target stimulus preceded or followed by the masking stimulus (fig. 4a) and calculated the response strength to the central target (see methods). The duration of the stimulus and mask varied to test the effect of the ON and OFF responses in masking. The surround mask alone did not evoke spike responses of the central neurons in agreement with neurophysiological observations. At the first layer, the surround mask did not significantly affect the ON and OFF responses to the central target (fig. 4b,c). Masking did, however, had a strong effect on the responses to the target of neurons in the second layer (fig. 4d,e). At short SOAs target responses were strongly suppressed (about 50%). For short mask durations, target responses at the different SOAs followed the characteristic U-shape (fig. 4d, dark blue line). For longer mask durations, the U-shape was split into two dips (fig. 4d, light blue lines). At a closer look the maximum dip was 25 ms, which was the duration of the target, before the onset and removal of the mask. This is indicated by the arrows in figure 4d, where the target responses are aligned to mask onset. We complemented this experiment by varying the target duration and maintaining the mask duration constant (fig. 4c,e). These results showed that for all target durations, masking was maximal when the time of target removal occurred at the same time as the presentation or removal of the mask. The results are explained by the transient surround inhibition, evoked by the presentation and removal of the surround mask. In particular, the coincidence of the mask responses with the target OFF responses seemed to contribute strongly to the suppressive effect (see insets fig. 4d,e). Weaker masking effects were also observed when the onset of the target and surround mask coincided (fig. 4e, open arrow).

**Figure 5:**
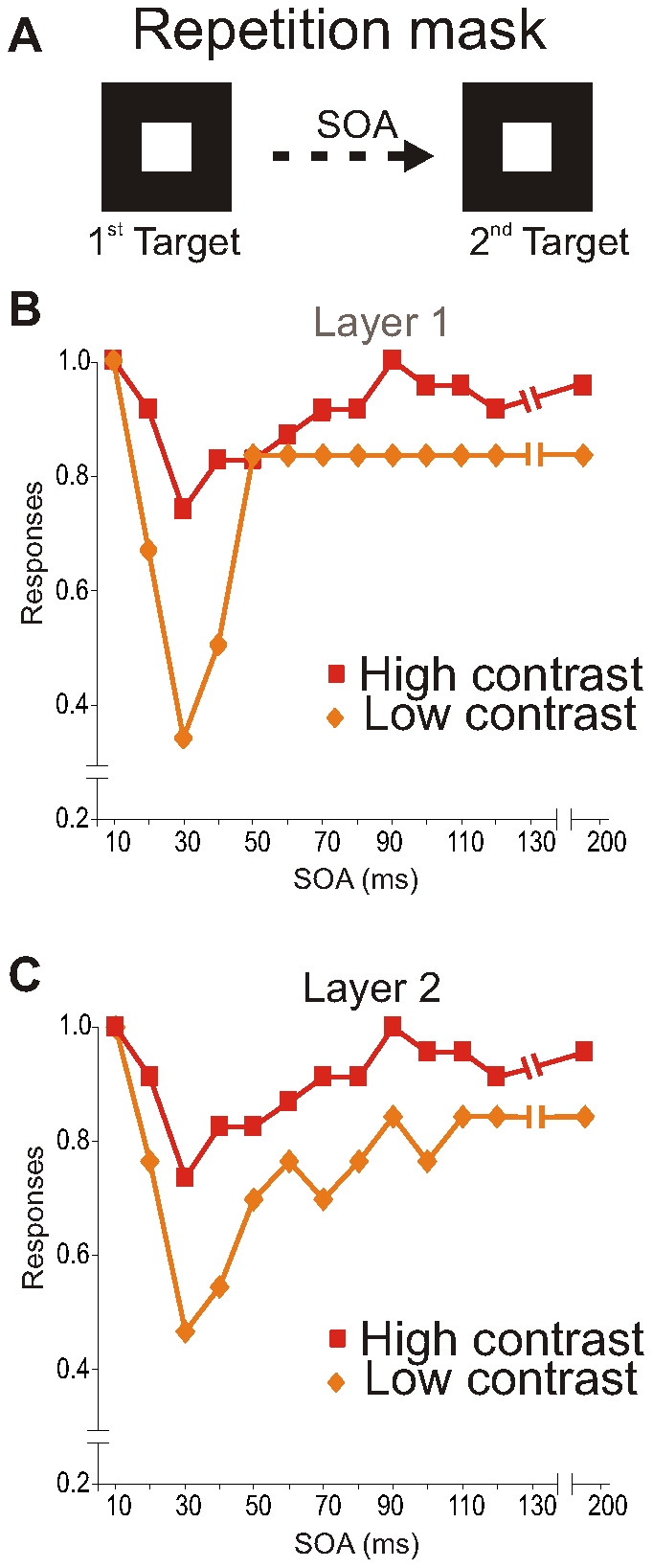
Repetition masking results. **A**: Squares indicate the first and the second target input in repetition masking experiment. **B,C**: Average spike responses, normalized to the target-only response, of neurons in the first (B) and second (C) layer to the second target stimulus. Time is from 1st target stimulus onset.

Finally, we tested the model for repetition masking (see methods), where the mask (or 2e target) is presented at the same location as the first target. We presented at different SOAs, a second target stimulus after the removal of the first target stimulus (fig. 5a). Both target stimuli were presented for 10 ms. and we calculated the responses to the second target. At short SOAs the responses to the second target were suppressed (∼50%) and recovered for longer SOAs (fig. 5b,c; orange lines). However, for the shortest SOA stimulus detection was normal, which is typical for repetition masking. When the second target had a higher contrast, the dip became less pronounced (fig. 5b,c; red lines).

## Discussion

Here we tested our computational spiking model that performs FG segmentation in a purely feed-forward manner [11], [22] in a backward masking paradigm. Despite its simplicity, the performance of the model bears similarities to neurophysiological findings on FG activity after backward masking. Testing the model in metacontrast and repetition masking tasks also show results that appear to be similar to behavioral and neural responses found under such masking conditions. Our masking results are explained by the spatiotemporal interference of surround inhibition by the mask.

### Figure-ground masking

In the pattern mask test, the results have a straightforward explanation. The mask produces strong surround inhibition to the layer-2 neurons in the Feat-1 channel reducing the transient target responses, especially when the SOA becomes shorter. In contrast, in the Feat-2 channel the pattern mask reduces the already strong surround inhibition to layer-2 neurons thereby enhancing the background responses. So figure and background responses become similar by shortening the SOA and thereby eliminating FG activity. These observations are similar to neuro-physiological findings in the primate visual cortex where FG activity gradually disappeared by backward masking [14]. Whether, the disappearance of FG activity in the visual cortex after backward masking also occurs by reducing figure responses and enhancing ground responses needs to be tested.

In contrast to a pattern mask, a uniform mask does not disrupt the FG signal nor does it impair visual perception of the figure [14]. In our study, responses in the first layer increased substantially for shorter SOAs because the uniform mask evoked responses at the background and figure locations in both Feat-1 and Feat-2 conditions. In the second layer, neurons in the Feat-2 condition remained quiescent because of the strong surround inhibition produced by the background and mask stimuli. In contrast, responses in the Feat-1 condition were not completely suppressed (see below). Thus FG activity remained even for the shortest SOA as previously observed in monkey visual cortex [14].

When the FG stimulus was presented for only 3 ms, FG modulation was still observed in the uniform masking experiment. This result is explained by the fact that layer-1 neurons were already slightly depolarized by the brief FG input (although spikes were not yet evoked). Consequently the mask input drove these layer-1 cells to earlier spiking than the neurons that were not stimulated by the FG stimulus (i.e. background in Feat-1 and figure region in Feat-2). As a consequence the produced surround inhibition by the mask arrived too late to immediately suppress the spiking of the central layer-2 neurons in Feat-1 condition. The network behaved completely different after a SOA of 5 ms; the condition which gave the strongest FG modulation in the uniform masking experiment. In this case, surround inhibition produced by the mask evoked rebound spiking [22] of central neurons in the Feat-1 condition, causing the observed enhanced figure responses. This finding emphasizes the utility of the Izhikevich neuronal type in contrasts to a simple IF neuron that is not capable of producing rebound spikes.

In the monkey visual cortex FG responses persist after uniform masking although for very short SOAs FG responses were weak [14]. Therefore our observations are similar to these findings. We also observed relatively stronger FG modulation after uniform masking than after pattern masking [14], but whether the strong FG activity in the visual cortex is caused by rebound spiking, as in our case, is not known. However, rebound spiking is a common neural phenomenon observed in the retina [24], [25], LGN [26]–[28] and visual cortex [29], may provide surface information [22], and may be critical to masking [18], [19].

### Metacontrast masking

Testing our model in metacontrast masking experiments showed that target responses followed the characteristic U-shape seen in perceptual masking studies [19]. For longer mask durations, the U-shape was split into two dips – a shape, which has also been observed for longer mask durations [19]. According to our data, masking was maximal when the time of target removal occurred at the same time as the presentation or removal of the mask. This was true for all target durations. Previous experiments have shown the importance of transient ON and OFF responses to the mask for the conscious perception of the target [18], [30], [31]. Our results explain such masking effect by the transient surround inhibition, evoked by the presentation and removal of the surround mask. In particular, the coincidence of the mask responses with the target OFF responses seemed to contribute strongly to the suppressive effect, in agreement with a neurophysiological report [18]. The minor suppression of the target ON response is in line with the observation of transient ON responses to an undetected target measured in low- [32] and high-level [33]–[35] areas after backward masking, and with target facilitation by masked priming [36]–[38].

### Repetition masking

In repetition masking, the second of two targets cannot be detected or identified when it appears close in time to the first one [17]. Previous studies have shown that optimal metacontrast-contrast masking only takes place when the target and mask are presented at the same location and share the same feature, e.g. orientation [39]–[41]. This indicates that the target stimulus and the mask stimulus activate the same set of neuronal cell population. We implemented this feature in our model by using the same Feat channel for both targets.

Our findings show that for short SOAs the responses to the second target were suppressed. However, for the shortest SOA stimulus detection was normal. This mimics the curious aspect of repetition masking, namely that targets presented very close together in time are not affected by the mask. Furthermore, in our study we observed that for a high contrast second target, the dip was less pronounced. This result is similar to the improved performance when the second target is made more salient [42]. So, our model behavior has a similar response pattern as detection performance found in repetition masking studies [17]. However, the timing of our model behavior is different to what is typically observed in human repetition masking studies. We observed a dip at 30 ms while in human masking studies a dip occurs around 50–300 ms. This may be related to the sheer difference in complexity between our model and the human visual system where processing times are longer. Even so, we believe that the important point is that the response modulations over time of our model show similar trends as human detection performance after masking. Our results of repetition masking are explained by the after hyper-polarization period, related to phase resetting curve [43] of the layer-1 neurons that prevents them to respond firmly to the second target. Phase resetting, which is influenced by feedforward and feedback projections is believed to be important for producing synchronous oscillations [44]–[46], see also [47]; a general mechanism of transient association between neuronal assemblies underlying sensory perception, see [48]. However, it remains to be tested whether in the visual system repetition masking results in a failure in phase resetting.

### Models on masking

By now, quite a few studies have investigated the neural basis of visual masking [14], [15], [18], [34], [35], [49]–[52]. In general, these experiments demonstrate that the responses to a target that has been effectively masked are suppressed, in particular the OFF responses at early visual stages [18]. Yet how this suppression comes about is debated and the theories concerning masking are controversial [12], [16], [49], [53].

Feedforward inhibitory models, e.g. [53] explain backward masking by asserting that the second stimulus exerts an inhibitory influence on the responses of a neuron evoked by the first stimulus, the target. To suppress the processing of the target in backward masking, the response to the mask needs somehow to catch-up with the target response. Inter-channel inhibition accounts of masking explain the temporal order by presuming the existence of two channels in visual processing. A fast channel used by the mask inhibits the slow channel which processes the target information. This model, however, fails to predict the different temporal features observed in forward and backward masking.

The lateral inhibitory model [19], [54] proposes a simple lateral inhibitory circuit to explain visual masking and argue that there is no reentrant feedback in masking [55]. Masking occurs when the transient spatiotemporal responses to the mask suppress the transient spatio-temporal responses of the target. Our findings on metacontrast masking support the lateral inhibitory model.

Another set of theories argue that the masking interferes with recurrent or reentrant processing [14], [49]. According to these models stimulus information flows from low to high visual levels and then back to the low ones. Only when the latter condition is properly met, the stimulus is sufficiently processed to allow for conscious detection. These models are partly based on the assumption that figure-ground activity critically depends on feedback. Our model, however, shows that FG does not critically depend on recurrent processing and that masking can disrupt feedforward FG activity.

In agreement with many other models of masking, see [37] for a review, our model findings highlight the importance of surround inhibition. In particular, our model emphasizes the role of inhibition evoked by the transient ON and OFF responses to the target and mask in visual detection; something that was predicted by the lateral inhibitory model [55]. The integration of surround information is under control of feedforward, local, and feedback projections [56]. Therefore, we propose that surround inhibition has a central function in visual detection and may bridge the different theories on masking. In addition, our findings emphasize that rebound spiking and phase resetting must also be considered in explaining visual masking.

### Feedforward, lateral and feedback connections in surround inhibition

Whether perceptual masking occurs, depends on the location of the mask relative to the target. As a general rule, to be effective the mask should be placed in close proximity of the target. A psychophysical report shows evidence for two distinct types of surround modulation; one narrowly tuned to iso-orientation and the other broadly tuned to cross-orientation [39]. These two surround types may relate to the proposed two separate neural mechanisms for surround suppression; one that arrives early consistent with a feedforward origin, and the other arrives late compatible with horizontal and feedback connections [57].

At all levels of the visual system, responses of neurons to stimuli presented in their receptive field are modulated by surround stimuli. In the macaque retina the suppressive field of the magno-cellular pathway is about four times the size of the excitatory field [58]. These retinal inhibitory effects are rapidly propagated to neurons in the LGN [59]–[61], where the influence of surround inhibition takes place at the very beginning of a stimulus response [61], [62]. This may lead to a reduction in the amount of excitatory potentials in the cortex [63]. In the cortex fast spiking neurons form an inhibitory network connected through electric synapses. Activation of these cells mediate strong and fast (<∼6 ms) thalamocortical feedforward inhibition that can shunt thalamocortical excitation [64], [65]. Thus in the visual cortex feedforward inhibition can suppress large regions and is fast where it can arrive even earlier to the target neuron than excitatory signals [66].

Another way to inhibit neural activity in a large cortical region is by long lateral or horizontal excitatory connections that activate local inhibitory cells. If, however, lateral connections are indeed the neural substrate of perceptual masking, the widespread inhibitory signal should arrive fast because masking depends strongly on the interference of the transient ON and OFF responses. The transfer of intra-cortical surround inhibition [67] and horizontal conduction velocities [56], [68] are however too slow to explain the suppression of the transients. Thus according to these data, lateral connections are unlikely to be the neural substrate for masking.

Feedback connections, which to V1 match the full spatial range of surround interactions, also contribute to surround suppression [56], [68]. These effects can be immediate as feedback from extra-striate cortex to V1 influences the earliest feedforward induced responses [69]. This means that transient stimulus responses are in fact a mixture of feedforward and feedback activity. This idea is in line with a recent transcranial magnetic stimulation study [70] that proposes an early overlap between recurrent and feedforward responses. Thus, feedback projections likely have a role in masking of transient responses by targeting directly and indirectly local inhibitory neurons. Further modeling studies however should reveal how visual masking occurs by including inhibitory cells. For instance is masking achieved by local acting inhibitory cells that receive widespread excitatory feedback projections or by local feedforward inhibition that is transmitted laterally within an inhibitory network?

## Methods

### Model architecture

In the figure-ground experiment, the model is composed of two feature channels each with two layers (fig. 1a) of NxN neurons of the Izhikevich type [71]. We used N = 64 but lower and higher values of N were also tested and did not critically affect model performance. The two separate feature channels represent two neuronal cell populations with opposite preference for a single feature. The channels are referred to as Feat-1 (central or figure stimulus) and Feat-2 (surrounding or background stimulus) condition. Because in the metacontrast- and repetition masking experiments there is only one target, the channels reflect the ON and OFF channels of the same feature where one channel detects the onset of the stimuli (target and mask) and the other the offset of the stimuli. The second layer integrates the input coming from the first layers of both channels (fig. 3a).

### Receptive fields

For all experiments, the excitatory feedforward projections from the stimulus input to the first neural layer and from the first to the second neural layer were retinotopic (point-to-point connections) where pixel/neuron N*_ij_* in the one layer connected only to neuron N*_ij_* in the next layer. Thus the excitatory part of a neuron's receptive field had size one. Neurons in the first neural layer did not receive inhibitory signals from the stimulus input. Each neuron in the second layer received inhibition from all neurons located in the preceding layer belonging to its feature channel (or from both channels in the metacontrast- and repetition masking experiments). Inhibition was achieved by assigning negative weights to the connections.

### Stimulus inputs

In the figure-ground experiments, the studied textured figures were two arrays of N×N pixels, with N as in the model. Input arrays were binary (0 or 1) corresponding to the preference for a single visual feature such as luminance, orientation, direction of motion, color etc. In other words, 1 stands for optimal tuning whereas 0 is the opposite. In the Feat-1 condition stimulus input was defined as an array of zeros except for the centre region of 16×16 pixels where the pixels had a value of 1, see also [11]. The other array was its binary complement, which represented the reverse preference of the visual feature. Together they formed the figure-ground texture [11], [20]. In the metacontrast- and repetition masking experiments, only the central target input was used for both channels. The homogenous texture was a matrix in which all pixels had a value of 1.

### Masking

In the figure-ground experiments, the pattern mask was a random binary (0 or 1) matrix of pixels and the uniform mask was a matrix in which all pixels had a value of 1. Masks were presented to both channels. In the metacontrast-masking experiment the mask stimulus was the complement of the target stimulus. This means that the target stimulus and the mask stimulus corresponded to the same preference for a single visual feature. Previous studies have shown that optimal metacontrast-contrast masking only takes place when the target and mask share the same feature, e.g. orientation [39]–[41]. The target and mask durations were varied (10, 25 and 50 ms for the target and 50, 100, 150 ms for the mask). In the repetition masking experiment the 2^nd^ target was identical to the target stimulus.

### Model dynamics

Cell dynamics is described by the spiking model of Izhikevich [71]

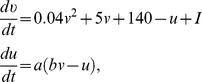
(1)supplemented with the after-spike reset rule
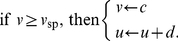
(2)

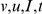
 are dimensionless versions of membrane voltage, recovery variable, current intensity and time. Further, *a* is a time scale for *u*, *b* measures the recovery sensitivity, *c* is the reset value for 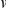, and *d* is the height of the reset jump for 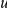. A capacitance factor C was chosen to be 1 and therefore omitted. For all our simulations *a* = 0.02, *b* = 0.25, *c* = −55, *d* = 0.05, and 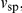 = 30. When dimensions are reintroduced, voltages are read in mV and time in ms. These values correspond to the phasic bursting type of the Izhikevich neuron.

As initial conditions at *t_0_* = 0 we set
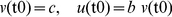
(3)for all the positions in our arrays (since we deal with two-dimensional objects, equations (1) and (2) are actually meant for 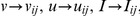 *i,j* = 1,…,N, and condition (3) is in fact applied to 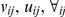. We used the Euler method with 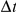 = 0.20 msec. The input current I in (1) is the result of summing different matrix contributions of the form
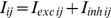
(4)where ‘exc’ stands for ‘excitatory’, ‘inh’ for ‘inhibitory’, and *i,j* are spatial indices.

Further,
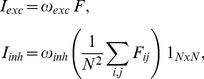
(5)

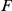
 is either the two dimensional stimulus input or the binary array defined by the presence of spikes, i.e., with ones where condition (2) is satisfied and zeros elsewhere. The 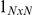 symbol denotes an NxN matrix containing just ones. Since excitatory receptive fields have size one, excitatory signals are point-by-point (retinotopic) copies of 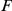 itself, multiplied by the corresponding weight. The inhibitory part, whose associate receptive field has the same size as 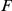, produces a spatially constant term –hence the 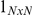 matrix- which is proportional to the normalized sum of all the F coefficients times the inhibitory weight. In our design, the used weights for all conditions were 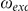 = 1 for the stimulus input to neural layer 1 and 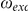 = 400, 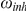 = −700 for the signals from neural layer 1 to neural layer 2. For the high-contrast condition in the repetition masking experiment the connections of the stimulus input to the first layer had 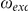 = 3.

### Calculating responses

To calculate the amount of figure-ground modulation we employed a modulation index (F–G), where F and G stand for the amount of spikes at the figure and ground regions, respectively during the first 50 ms. The figure (background) responses from the two central (surround) regions of both feature channels were averaged. In the metacontrast- and repetition masking experiments, responses were calculated over a time window of 100 ms. starting from target (or 2^nd^ target in repetition masking) onset.
